# Decreased Tumor Progression and Invasion by a Novel Anti-Cell Motility Target for Human Colorectal Carcinoma Cells

**DOI:** 10.1371/journal.pone.0066439

**Published:** 2013-06-03

**Authors:** Qunyan Jin, Guangming Liu, Phillip P. Domeier, Wei Ding, Kathleen M. Mulder

**Affiliations:** 1 Department of Biochemistry and Molecular Biology, Penn State Hershey College of Medicine, Hershey, Pennsylvania, United States of America; 2 Department of Pediatrics, Penn State Hershey College of Medicine, Hershey, Pennsylvania, United States of America; Ghent University, Belgium

## Abstract

We have previously described a novel modulator of the actin cytoskeleton that also regulates Ras and mitogen-activated protein kinase activities in TGFβ-sensitive epithelial cells. Here we examined the functional role of this signaling regulatory protein (km23-1) in mediating the migration, invasion, and tumor growth of human colorectal carcinoma (CRC) cells. We show that small interfering RNA (siRNA) depletion of km23-1 in human CRC cells inhibited constitutive extracellular signal-regulated kinase (ERK) activation, as well as pro-invasive ERK effector functions that include phosphorylation of Elk-1, constitutive regulation of c-Fos-DNA binding, TGFβ1 promoter transactivation, and TGFβ1 secretion. In addition, knockdown of km23-1 reduced the paracrine effects of CRC cell-secreted factors in conditioned medium and in fibroblast co-cultures. Moreover, km23-1 depletion in human CRC cells reduced cell migration and invasion, as well as expression of the ERK-regulated, metastasis-associated scaffold protein Ezrin. Finally, km23-1 inhibition significantly suppressed tumor formation in vivo. Thus, our results implicate km23-1 as a novel anti-metastasis target for human colon carcinoma cells, capable of decreasing tumor growth and invasion via a mechanism involving suppression of various pro-migratory features of CRC. These include a reduction in ERK signaling, diminished TGFβ1 production, decreased expression of the plasma membrane-cytoskeletal linker Ezrin, as well as attenuation of the paracrine effects of colon carcinoma-secreted factors on fibroblast migration and mitogenesis. As such, km23-1 inhibitors may represent a viable therapeutic strategy for interfering with colon cancer progression and invasion.

## Introduction

Human colorectal cancer (CRC) is one of the most common malignancies, with distant metastases representing the greatest threat to patient survival [1]. Prior to the development of frank metastatic lesions, cancer cells exhibit properties consistent with a propensity to migrate and invade into surrounding tissues and distal organs [2], [3]. Various cellular events are known to be associated with this increased potential for malignant cells to spread to local and distant sites. Among these events are elevated expression or activity of signaling components and cellular scaffolds [2], [4]. However, a complete understanding of the highly integrated network of signaling pathways and complexes relevant to the cell migration and invasion process is still lacking and often depends on the tissue of origin, as well as on the precise combination of oncogenically active alterations that prevail.

A role for abnormal ERK signaling in human cancer, due to is its positive influence on cellular survival and proliferation, is well-established [5], [6]. However, the ERK pathway also controls tumor cell migration, invasion, and progression [5], [7], . Aberrantly high ERK activity is often caused by Ras/MAPK pathway genes being frequently mutated in human cancers, making them the target of numerous anticancer therapeutic strategies [5], [6]. For example, nearly 50% of colon cancers harbor activating mutations in KRAS and 5–18% display activating BRAF mutations [10], [11]. Moreover, these mutations in the K-Ras and B-Raf signaling intermediates occur in a mutually exclusive manner [10].

In addition to constitutive ERK activity, downstream effectors may also be associated with a pro-migratory phenotype of cancer cells. For example, Ets family members such as Elk-1 represent a major class of transcription factors activated by the ERK cascade, which can induce changes in cell migration, as well as in expression of activator protein-1 (AP-1) transcription factors [12], [13]. AP-1 components, themselves, also regulate cell motility and invasion in various malignant epithelial cells, including colon carcinomas cells [14], [15]. In addition, other invasion-related changes in gene expression are mediated by ERK and AP-1 pathway activation. For example, ERK/AP-1 signaling is required for transactivation of the VIL2 gene promoter [16], leading to Ezrin expression, the up-regulation of which has been associated with tumor invasion and metastasis of CRC cells [17]. While Ezrin facilitates signaling by adhesion molecules and growth factors, it is also an actin cytoskeletal linker critical for the dynamic regulation of cell motility and invasion [18], [19]. Thus, constitutive ERK activity may also influence cytoskeletal-scaffolding factors that play a pro-migratory role during invasion.

Uncontrolled activation of ERK signaling is also associated with the constitutive production of TGFβ, a known inducer of invasive phenotype in several cancer types, including colon cancer cells [20], [21]. While TGFβ is a natural pleiotropic growth factor that has the capacity to regulate diverse biologic processes for a variety of cell types, tumor cells lose responsiveness to the negative growth control signals of TGFβ [22], [23]. The escape of the cancer cells from TGFβ-mediated growth control is often associated with mutations in the type II TGFβ receptor (RII) gene and/or with alterations in TGFβ signaling pathways [23], [24], [25]. However, cancer cell-secreted TGFβ can still stimulate tumor progression and invasion through its paracrine effects in promoting angiogenesis, inhibiting immune surveillance, and up-regulating extracellular matrix components in the tumor microenvironment [23], [26], [27]. Further, increased TGFβ signaling in tumor cells that retain TGFβ responsiveness can induce epithelial-mesenchymal transition (EMT), often associated with a pro-migratory phenotype [23], [26], [28]. However, EMT is not required for invasion of CRC cells, since a major subtype of human CRC associated with disease progression and recurrence does not undergo EMT [29]. Nevertheless, specifically blocking the signaling pathways that mediate production of TGFβ [20], to inhibit its pro-oncogenic and pro-migratory effects, represents an important therapeutic strategy for human cancers.

With regard to these regulators of the pro-migratory and pro-invasive phenotype associated with malignant progression, we have reported that km23-1 regulates RhoA activity and actin modulating proteins, which establish km23-1 as an anti-motility target [30]. km23-1 (also called DYNLRB1/mLC7-1/robl-1/Dnlc2a/DYRB1) was originally identified in a novel screen for components activated by the TGFβ receptor complex, but it is also a light chain of the motor protein dynein [31]. NMR and X-ray crystal structure analyses demonstrated that km23-1 exists in cells as a stable homodimer, structurally unrelated to other dynein light chains (Tctex1/DYNLT and LC8/DYNLL) [32], [33]. However, as for these other dynein light chain families, km23-1 is multifunctional and can regulate both dynein-dependent and -independent functions [34], [35]. Moreover, km23-1 has been shown to mediate specific TGFβ signaling events and downstream responses [31], [34], [36]. For example, km23-1 is required for TGFβ1 production through dynein-independent Ras/ERK/Jun N-terminal kinase (JNK) pathways in TGFβ-sensitive epithelial cells [35]. This multifunctional regulator also plays a critical dynein-dependent role in Smad2 signaling in early endosomes [34], and it is regulated by protein kinase A (PKA) activation [37]. Depletion of km23-1 not only reduces fibronectin (FN) expression, but also RhoA activity [30], further demonstrating the critical nature of km23-1's regulatory functions. Here we describe a novel inhibitory effect on tumor progression, migration, and invasion upon depletion of km23-1. Further, our results demonstrate that km23-1 may be a novel anti-metastatic target for CRC, showing for the first time that km23-1 regulates constitutive ERK activation, the pro-migratory effects of high Ezrin expression, TGFβ1 production, and the paracrine effects of CRC cell-secreted factors. In addition, km23-1 silencing reduced Ezrin expression in human CRC cells that have invaded through a three-dimensional (3D) matrix.

## Materials and Methods

### Reagents

Anti-dynein intermediate chain (DIC) (MAB 1685) was from Chemicon (Temecula, CA), and anti-c-Jun (sc-45×), anti-c-Fos (sc-52×), and anti-TGFβ1 were from Santa Cruz Biotechnology (Santa Cruz, CA). Anti-phospho-p44/42MAPK (phospho Erk1/2), anti-p44/42 MAPK (Erk1/2), anti-phospho-Elk-1, anti-Elk-1, and anti-Ezrin (#3145) were from Cell Signaling Technology (Danvers, MA). McCoy's 5A modified medium was from Invitrogen (Grand Island, NY, USA). The piLenti negative control (NC) siRNA-GFP (LV015-G) and the piLenti km23-1 siRNA-GFP set (i006555) were from Applied Biological Materials Inc. (Canada). Other chemicals were from Sigma unless otherwise indicated.

### Cell culture

RKO, HCT116, and CBS human CRC cells [38] were routinely cultured in McCoy's 5A medium, supplemented with amino acids, pyruvate and antibiotics (streptomycin, penicillin), with 10% fetal bovine serum (FBS). Cells were routinely screened for mycoplasma using Hoechst 33258 staining.

### Real-time quantitative PCR

RNA was isolated from RKO stable tranfectants using TRIzol Reagent by Invitrogen Corporation (Carlsbad, CA). Real-Time Quantitative PCR was performed using SYBR Green I agent by QIAGEN Inc. (Valencia, CA) as described previously [39]. 18S rRNA was used as an internal control. Highly purified primers (sense: CATCAAGAGCACCATGGACAA, antisense: CAGGATGAAGCTGTGCATGAG) for km23-1 were used.

### Generation of RKO CRC cell clones stably expressing small hairpin RNAs (shRNAs)

The sense strand of the hairpin km23-1 siRNA, corresponding to nucleotides 251–271 of the human km23-1 coding region (5′-AAGACTATTTCCTGATTGTGA-3′), was inserted into the pRNATin-H1.2/hygro siRNA vector (GenScript, Piscataway, NJ) according to the manufacturer's instructions. A scrambled siRNA control (NC siRNA) was also provided by the company. These pRNATin-H1.2/hygro siRNA vectors were transfected into RKO cells using Lipofectamine 2000 (Invitrogen) according to the manufacturer's protocol. The empty vector (EV), NC siRNA, and km23-1 siRNA (km23-1 siRNA clone #1 and clone #5) RKO stable transfectants were prepared, characterized, and routinely maintained in growth medium with 100 µg/ml of hygromycin B as described previously [35].

### Preparation of lentiviruses, siRNA production, and transient cell infections

Lentiviruses were generated by transfecting either the pilenti-NC siRNA-GFP or the piLenti km23-1 siRNA-GFP set [which contains four different pilenti-km23-1 siRNA-GFP plasmids (ABM # i006555)], together with packaging plasmids (LV003, Applied Biological Materials Inc) into 293T cells using lipofectamine 2000 transfection reagent (Invitrogen) according to the manufacturer's protocol. Viral supernatants were harvested at 48 h after transfection and were filtered through a 0.45-mm filter. For Ezrin experiments, HCT116 and CBS cells were infected with lentiviral particles expressing the indicated plasmids in the presence of 8 µg/ml polybrene according to the manufacturer's instructions. 24 h after infection, cells were harvested and subjected to Western blotting analyses.

### Generation of stable lentivirus-expressing cell lines

HCT116 and CBS cells stably expressing the lentivirus siRNAs indicated above were generated by lentiviral transduction in the presence of 8 µg/mL polybrene according to the manufacturer's instructions, followed by selection with 2 µg/ml of neuromycin over 2 weeks. Cells were routinely maintained in growth medium with 1 µg/ml of neuromycin.

### Chromatin immunoprecipitation (ChIP) assays, Westerns, luciferase reporter assays, quantitation of TGFβ1 by enzyme-linked immunosorbent assays (ELISAs)

Chromatin immunoprecipitation (ChIP) assays, Westerns, luciferase reporter assays, quantitation of TGFβ1 by enzyme-linked immunosorbent assays (ELISAs) were performed as described previously [20], [40].

### Transwell migration assays

Transwell migration assays were performed as described previously [20], [40]. Briefly, NIH3T3 cells were seeded in polycarbonate membrane filter inserts (8.0 µm pore size) in 6-well Transwells (Corning Life Sciences, Acton, MA) at 5×10^5^ per well with 2 ml medium in the upper insert chamber and 1 ml medium in the lower chamber. 12 h after the cells were seeded, the medium in both upper insert and lower chamber was removed. Conditioned medium (CM) (2 ml) was collected from the RKO cell cultures and their stable transfectants, and was diluted with serum-free (SF) DMEM at a ratio of 1∶1, prior to adding to the upper insert well. SF DMEM (1 ml) was also added to the lower chamber and the cells were grown for an additional 48 h. NIH 3T3 cells that had migrated into the lower chamber through the 8.0 µm pore membrane were counted according to the manufacturer's instructions.

### Co-culture of RKO and NIH3T3 cells and mitogenesis assays

Co-culture of RKO and NIH3T3 cells and mitogenesis assays were performed as described previously [20], [40]. 12-well Transwells with polyester membrane filter inserts (0.4 µm pore size) were used for the co-culture experiments, because cells cannot migrate through filters of this pore size. However, the medium can be exchanged between the RKO and 3T3 cells under these conditions. Briefly, RKO cells and their stable transfectants were seeded in the upper insert at 10^5^ cells per well with 0.5 ml normal growth medium. NIH3T3 cells were seeded in lower chamber at 10^4^ cells per well with 1 ml DMEM containing 10% FBS. The medium were then changed to SF DMEM in the lower chamber for the NIH3T3 cells, and to normal growth medium in the upper insert for the RKO cells, at 12 h after the cells were seeded. After a 36 h incubation in SF medium, [^3^H]-thymidine was added to the medium in the lower chamber for NIH3T3 cells, and the thymidine incorporation assay was performed to assess NIH3T3 cell mitogenicity in response to RKO cell co-culture.

### In vitro migration assays

The Costar Transwell System (8-µm pore size polycarbonate membrane, 6.5-mm diameter, Corning, Inc., Corning, NY) was used to evaluate cell migration. Briefly, stable HCT116 cells (4×10^5^/well) were suspended in 500 µl of SF medium and seeded in the upper well of the Costar Transwell System. 500 µl of 10% FBS medium was added to the bottom wells of the plate. Thereafter the plates were incubated for 24 h at 37°C in 5% CO_2_. After incubation, non-migrated cells were removed from the upper surface of the chamber with a cotton swab. Migrated cells were stained with DAPI for nuclear DNA in an immunofluorescence assay. The number of migrating cells from at least seven fields of each of three separate membranes was counted under a fluorescence microscope using a 10× objective [41].

### Matrigel invasion assays

Matrigel invasion assays were analyzed in a BioCoat™ Matrigel™ Invasion Chamber (Becton-Dickinson, Bedford, MA, USA) according to the protocol provided by the manufacturer. Briefly, RKO stable transfectants (1×10^5^ cells/ml) were suspended in McCoy-BSA (0.1%) medium and seeded onto Matrigel-coated Transwell filters (8-µm pore size) in BioCoat Matrigel invasion chambers. Epidermal growth factor (EGF) was diluted into McCoy-BSA medium at 20 ng/ml and added to the lower well. Chambers were incubated at 37°C for 24 h, after which filters were removed, fixed, and stained with 0.2% (vol/wt) crystal violet. After two washes with distilled water, the chambers were allowed to air dry. The number of invading cells from at least seven fields of each of three separate membranes was counted under the light microscope using a 10× objective [42].

### Immunofluorescence analyses in the inverted invasion assay

Immunofluorescence analyses in the inverted invasion assay were performed as described previously [43] with slight modifications. In brief, HCT116 stable cells that migrated through the lower membrane in the Matrigel invasion assay were fixed in 4% formaldehyde for 20 min followed by permeabilization in 0.1% Triton X-100 for 5 min and blocking in 5% BSA/PBS for 1 h. Subsequently, these cells were incubated with Ezrin antibody (1∶100) at 4°C overnight. After cells were washed extensively in blocking buffer, 2 µg/ml cy3-conjugated goat anti-rabbit IgG secondary antibody was added in blocking buffer for 1 hour at room temperature (red). 4′,6-Diamidino-2-phenylindole (DAPI) staining designates individual cells. Duplicate fields are shown for each condition.

### Cell growth assays

For RKO stable clones, cells were plated at a density of 2.0×10^3^ cells/well in 6-well plates and cell number was determined by hemocytometer counting on the indicated days following plating. For HCT116 and CBS stable cell pools, cell viability was assessed using the cell proliferation kit II (XTT; Roche Molecular Biochemicals, Indianapolis, IN, USA) according to the manufacturer's protocol. Briefly, cells were plated at a density of 7.5×10^3^ cells/well in 96-well plates. 48 h after plating, cells were incubated with the XTT dye at 37°C for 1 h and the absorbance was read at 490 nm. Analyses were performed in triplicate and viability is expressed as a percentage of control cells.

### Tumorigenicity Studies

Six-week-old Balb/c athymic female mice were purchased from Charles River Laboratories (Wilmington, MA). The use of athymic nude mice and their treatment was approved by the Institutional Animal Care and Use Committee (IACUC), Penn State Hershey College of Medicine, and all the experiments were carried out in strict compliance with their regulations. NC-siRNA-RKO cells and km23-1-siRNA RKO clone #1 and #5 cells (5×10^6^) were inoculated subcutaneously behind the right anterior forelimb of the mice (5 mice per group) and tumorigenicities were determined [40].

### Statistical analysis

Statistical analysis was by Student's t test unless otherwise indicated. Triplicate samples were analyzed and mean ± SE plotted unless otherwise indicated.

## Results

### Depletion of km23-1 reduces constitutive activation of ERKs in human CRC cells

**Figure 1:**
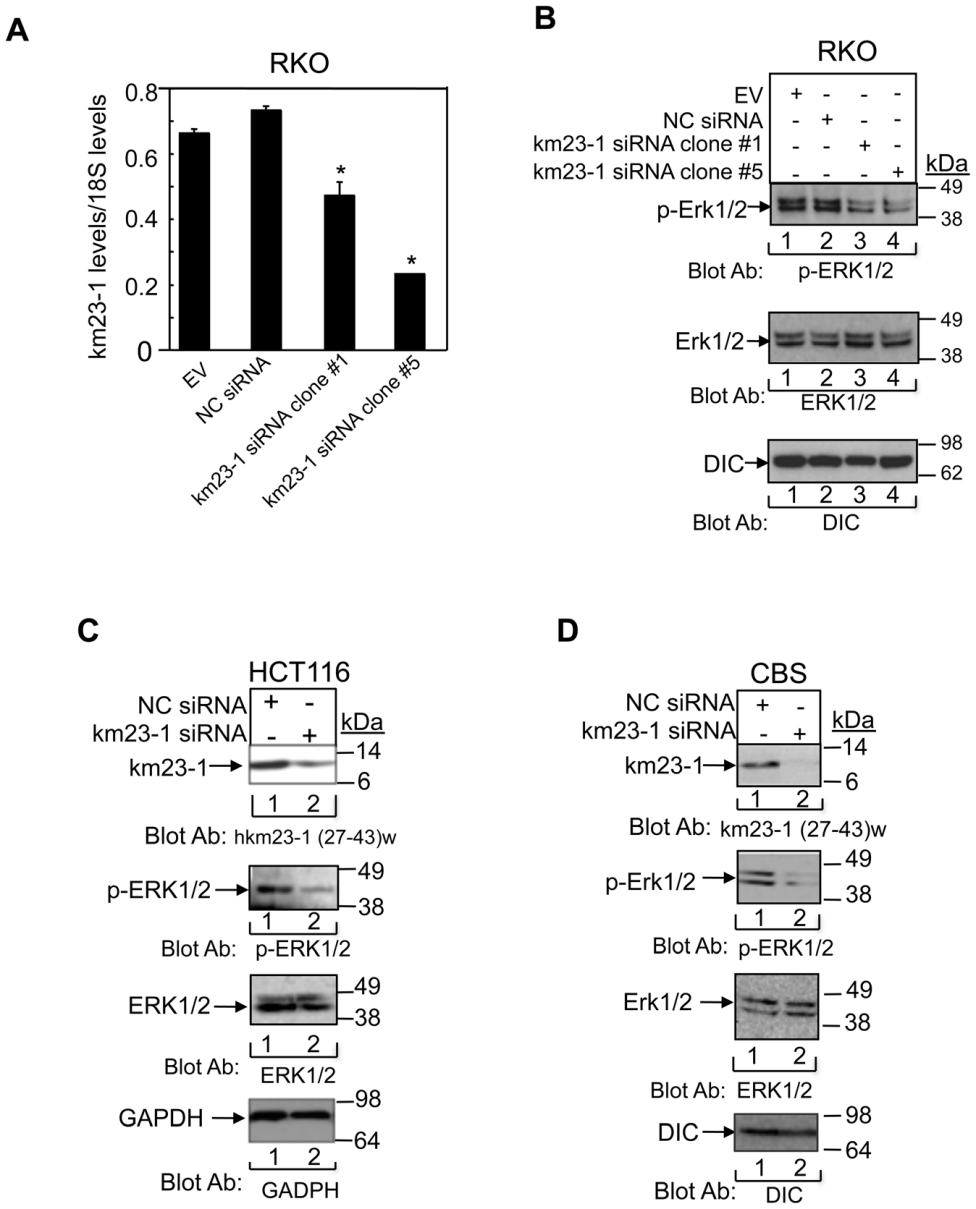
Depletion of km23-1 blocks constitutive ERK activation in human CRC cells. **A.** EV, NC siRNA, and km23-1 siRNA stable transfectants (clones 1 and 5) were used to isolate RNA. RT-PCR was performed and products were analyzed as described in the “Materials and Methods.” The data plotted are the mean ± SE of three independent experiments. *p<0.01 compared to the NC siRNA. **B:** Western blotting of phospho- and total protein expression levels for ERK1/2 in RKO human CRC cells. **Bottom**, DIC protein was assessed as a loading control. Data are representative of three independent experiments. **C**: Western blotting of phospho- and total protein expression levels for ERK1/2 in HCT116 cells stably transduced with the lentiviral particles described in the “Materials and Methods.” Top, confirms knockdown of endogenous km23-1. **Bottom**, GAPDH protein was assessed as a loading control. Data are representative of three independent experiments. **D:** CBS cells were infected with either pilenti-NC siRNA-GFP or pilenti-km23-1 siRNA-GFP pools. 24 h after infection, Western blotting was performed using the indicated antibodies. **Top**, confirms knockdown of endogenous km23-1. **Bottom**, DIC protein was assessed as a loading control. Three independent experiments were performed and representative figures are shown.

Our previous results have shown that km23-1 is required for RhoA activity and cell migration, via its association with key proteins involved in actin-based cell motility and modulation of the actin cytoskeleton [30]. Therefore, our previous results suggest that km23-1 may play a crucial role in the motility of human CRC cells [30]. In addition, sustained ERK activation can be required for cell migration induced by a variety of growth factors and cytokines [44]. Since RKO cells harbor a V600E BRAF mutation [45], resulting in constitutive ERK activation, it was of interest to determine whether km23-1 knockdown could inhibit the constitutive ERK activity in this model system. The pRNATin-H1.2/hygro km23-1 siRNA and the relevant NC siRNA sequences have been described previously [34], [36]. Further, we have developed RKO cell clones (#1, #5) stably expressing km23-1-specific siRNA and confirmed km23-1 depletion by Western blot analysis of protein lysates isolated from the stable transfectants as described previously [35]. We further confirmed the efficiency of km23-1 silencing in RKO cells at the mRNA level. The stable transfectants were harvested for RNA isolation and RT-PCR was performed using primers specific for km23-1 (Fig. 1A). Next, we performed phospho-blotting for ERK1/2 in these stable RKO human CRC cells. As shown in Fig. 1B, the cells stably transfected with EV and NC siRNAs displayed constitutive phosphorylation of ERK1/2 (lanes 1–2). In contrast, in RKO cells stably transfected with km23-1 siRNA, the phosphorylation of ERK1/2 was significantly suppressed, whereas there was no effect on total ERK1/2 expression levels (lanes 3–4). Thus, km23-1 inhibition attenuated ERK1/2 activation in RKO human CRC cells. Similarly, knockdown of km23-1 suppressed ERK1/2 phosphorylation in both HCT116 (Fig. 1C) and CBS (Fig. 1D) human CRC cells. These cells were chosen because they harbor G13D and G12D KRAS mutations, respectively [24], which ultimately result in constitutive ERK activation. In addition, HCT116 cells are TGFβ receptor RII-deficient [25]. Taken together, our results demonstrate that depletion of km23-1 expression inhibits ERK activation in three different cancer cell lines with constitutively activated ERK.

### Depletion of km23-1 inhibits TGFβ1 promoter transactivation, TGFβ1 secretion, c-Fos-DNA binding at the proximal AP-1 site of the human TGFβ1 promoter, and Elk-1 activation in RKO human CRC cells

**Figure 2:**
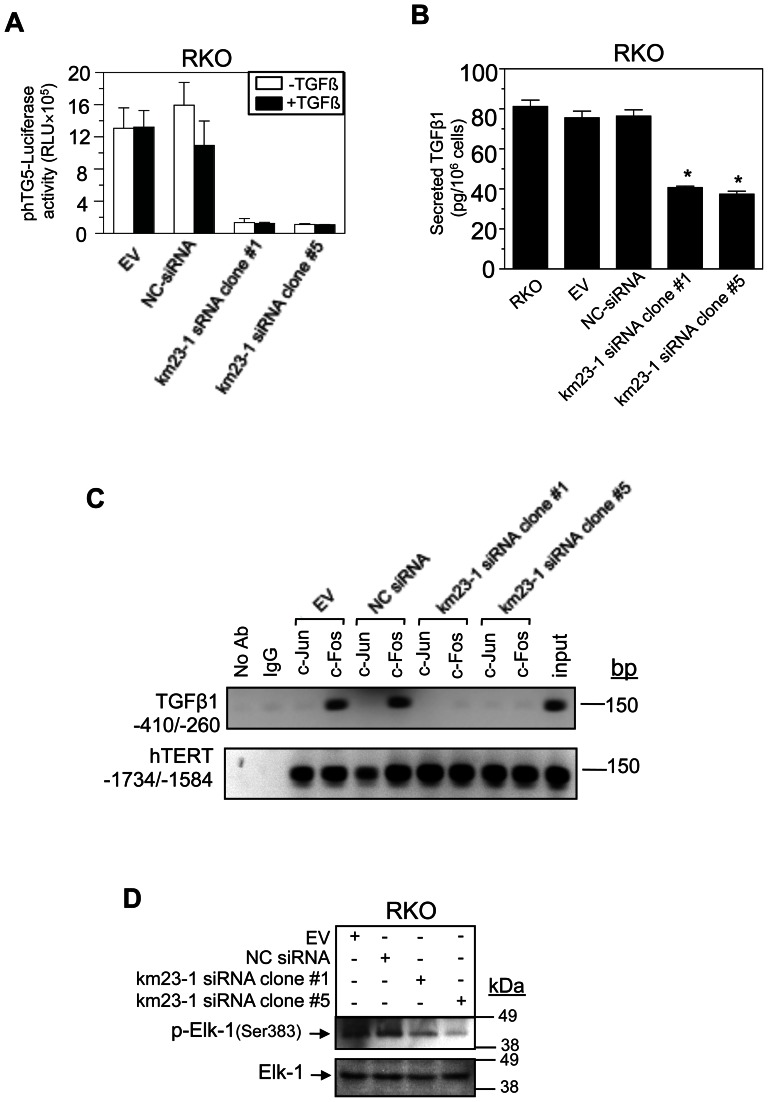
Depletion of km23-1 in RKO human CRC cells inhibits transactivation of the proximal AP-1 site in the TGFβ1 promoter, TGFβ1 secretion, c- Fos-DNA binding to the TGFß1 promoter, and Elk-1 activation. **A:** RKO stable transfectants were transiently transfected with phTG5-Lux and luciferase assays were performed as described previously [20], [40]. Data are representative of three independent experiments. **B:** TGFβ1 concentrations in CM from RKO stable transfectants were measured by ELISAs as described previously [20], [40]. *p<0.01 compared to the NC siRNA. **C:** ChIP assays were performed [20], [40] using either the human TGFβ1 promoter region from −410 to −260 (upper panel) or the hTERT promoter region from −1734 to −1584 (lower panel) with the indicated antibodies. Input: Equal samples of chromatin DNA without prior immunoprecipitation. **D:** Western blotting of phospho- and total protein expression levels for Elk-1 in RKO human CRC cells. Three independent experiments were performed and representative figures are shown.

The ERK and JNK pathways are known to be required for TGFβ1 production, which is an important biological response of TGFβ [46]. Specifically with regard to human CRC, we have shown that TGFβ1 production requires c-Fos activation, which can be targeted to decrease tumor progression in vivo [40]. Since we have now found that knockdown of km23-1 also reduced sustained ERK activation, we examined whether TGFβ1 promoter activity downstream would also be repressed by km23-1 knockdown. The RKO human CRC cells were used for these studies because they have alterations in TGFβ RII receptors [25], resulting in constitutive TGFβ1 production, not being regulated by either autocrine or exogenous TGFβ1 [22], [47]. Moreover, the TGFβ1 production pathway in this CRC cell model system has been well-characterized [20]. The stable RKO transfectants were transiently transfected with phTG5-lux, previously constructed to measure transcriptional activation of the AP-1 site in the human TGFβ1 promoter [48], and luciferase activities were determined. As shown in Fig. 2A, exogenous TGFβ1 did not significantly alter phTG5-Lux activity for any of the samples, as expected. Further, EV and NC-siRNA cells revealed levels corresponding to constitutive activation of the phTG5-lux reporter in RKO human CRC cells. In contrast to these cells, phTG5 activities in km23-1 siRNA clone #1 and #5 were significantly decreased, demonstrating that km23-1 is required for transactivation of the human TGFβ1 promoter site required for TGFβ1 expression in RKO cells.

Since km23-1 was required for TGFβ1 promoter transactivation, it was conceivable that TGFβ1 secretion into the conditioned medium of km23-1siRNA-RKO cells could also be attenuated. As shown in Fig. 2B, quantitative determination of TGFβ1 in CM by ELISAs revealed significantly suppressed levels in km23-1 siRNA clone #1 and #5. In contrast, neither EV nor NC-siRNA cells displayed differences in TGFβ1 production relative to the parental RKO cells. Thus, km23-1 is required for TGFβ1 secretion in RKO human CRC cells.

We have previously shown that the TGFβ1 production pathway is unique in human CRC cells, being regulated by c-Fos-dependent TGFβ1 promoter activation in a constitutive, ligand-independent manner [20], [40]. This is in contrast to TGFβ autoregulation of the relevant promoter site in TGFβ-sensitive epithelial cells, which utilizes a JunD- and Fra-2-dependent mechanism [46]. Since km23-1 KD inhibited TGFβ1 promoter transactivation and TGFβ1 secretion in RKO cells, we examined the binding of c-Fos and c-Jun to the proximal AP-1 binding site in the human TGFβ1 promoter by ChIP assays (Fig. 2C). This motif (−362 to −335) in the TGFβ1 promoter is critical for mediating TGFβ1 expression in human cells specifically (ie, compared to mouse cells) [46], [48]. As expected for RKO cells [20], EV and NC siRNA cells displayed c-Fos, but not c-Jun, binding. In contrast, c-Fos binding to the AP-1 site in the TGFβ1 promoter was completely inhibited in km23-1-siRNA-RKO clone #1 and #5 (Fig. 2C, upper panel). For an additional positive control, we used the hTERT promoter (Fig. 2C, lower panel). As expected, both c-Jun and c-Fos bound to the hTERT promoter [49], and km23-1 depletion had no effect on this binding. Thus, km23-1 is required for c-Fos binding to the TGFβ1 promoter site that mediates TGFβ1 expression in RKO human CRC cells.

It is known that ERK activation leading to c-Fos transcriptional effects is often mediated through activation of the Ets-domain transcription factor Elk-1 [12], [50]. ERK1/2 can efficiently phosphorylate Elk-1 at serine 383 and cause transcriptional activation of components such as c-Fos [50]. Hence, we investigated the effects of km23-1 depletion on activation of Elk-1 in the RKO stable transfectants by phospho-blotting (Fig. 2D). In contrast to the controls (EV, NC), blockade of km23-1 expression suppressed Elk-1 phosphorylation at serine 383 in clones 1 and 5, but had no effect on total Elk-1 expression. Thus, km23-1 is required for Elk-1 activation in RKO human CRC cells. Collectively, km23-1 depletion reduced Elk-1 activation, the constitutive regulation of c-Fos-DNA binding, TGFβ1 promoter transactivation, and TGFβ1 secretion in human CRC cells.

### Knockdown of km23-1 suppresses the paracrine effects of TGFβ1 on fibroblast migration and mitogenesis

**Figure 3:**
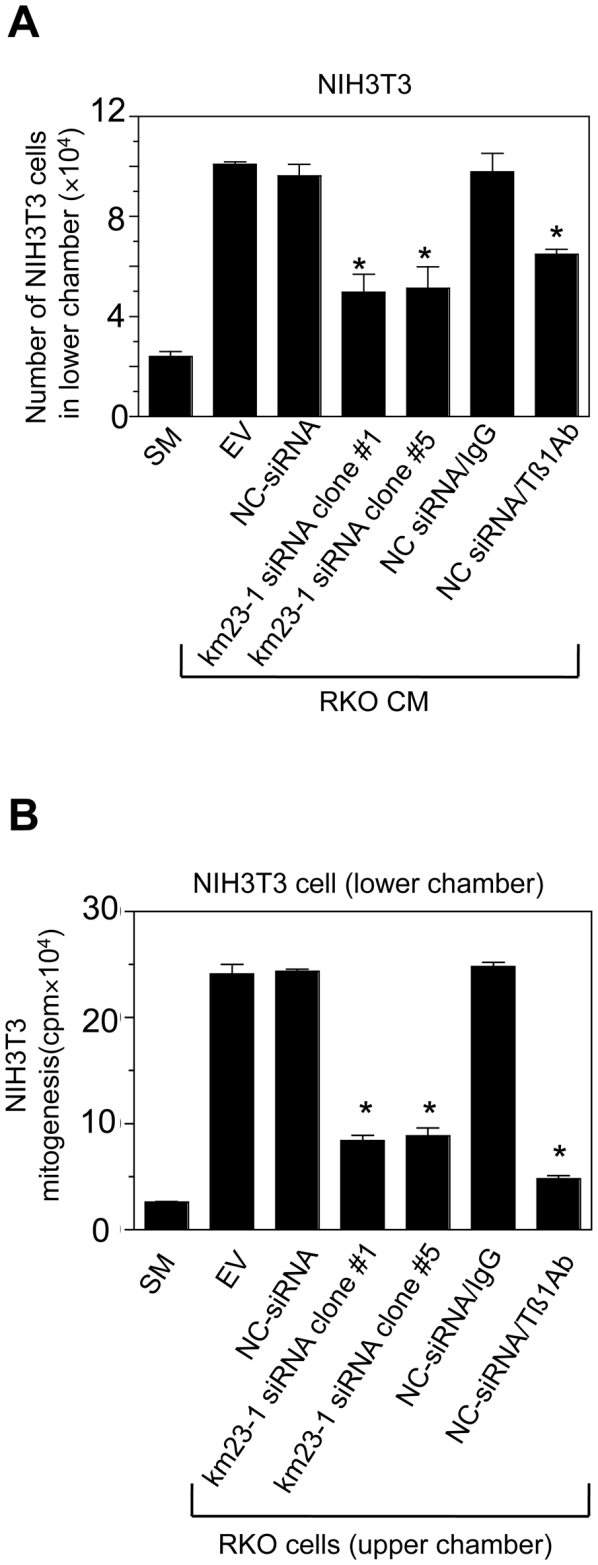
km23-1 regulates the paracrine effects of active TGFβ1 on NIH3T3 cell migration and mitogenesis. **A:** NIH3T3 fibroblasts were plated onto polycarbonate membrane filter inserts (8.0 µm pore size) in 6-well Transwells and CM from RKO cells and their siRNA stable transfectants were added to the NIH3T3 cells as described in “Materials and methods.” Cells migrating into the lower chambers were counted. SM: supplemented McCoy's (SM) medium; EV: CM from RKO-EV cells; NC siRNA: CM from NC siRNA-RKO cells; km23-1-siRNA clone #1: CM from km23-1-siRNA-RKO clone #1; km23-1-siRNA clone #5: CM from km23-1-siRNA-RKO clone #5; NC siRNA/Tβ1Ab: CM from NC-siRNA cells that had been incubated with a neutralizing anti-TGFβ1 antibody (30 µg/ml); Ctrl/IgG: CM from Ctrl with IgG. Data plotted are the mean ±SE of triplicate wells from a representative experiment (n = 3). *p<0.01 compared to either NC-siRNA or NC-siRNA/IgG. **B:** NIH3T3 cells were plated in the lower chamber and made quiescent as described in the “Materials and methods.” RKO cells and their siRNA stable transfectants were then plated onto polyester membrane filter inserts (0.4 µm pore size) in 12-well Transwells. Mitogenesis assays were performed as described in “Materials and methods” to assess the effect on NIH3T3 cell mitogenesis after co-culture with RKO stable transfectants. Data plotted are the mean ±SE of triplicate wells from a representative experiment (n = 3). *p<0.01 compared to the NC-siRNA or NC-siRNA/IgG.

The parental RKO cells contain TβRII mutations and consequently, do not display autocrine regulation or response to exogenous TGFβ [20]. However, the TGFβ1 they secrete might create a pro-oncogenic microenvironment more advantageous for tumor growth [40] by affecting stromal cells, such as fibroblasts. Since NIH3T3 fibroblasts respond to TGFβ with increased cellular migration and mitogenicity, they provide a useful culture model to examine the paracrine effects of RKO cell-secreted factors [20]. To determine whether knockdown of km23-1 would block the paracrine effects of tumor cell-secreted TGFβ1, CM from the RKO stable transfectants was examined for effects on the cell migration of NIH3T3 fibroblasts. As expected, CM from EV- or from NC-siRNA cells stimulated the migration of the NIH3T3 cells, compared to fibroblasts grown in supplemented McCoy's 5A (SM) medium only (Fig. 3A). In contrast, CM from cultures of km23-1-siRNA clone #1 or #5 cells significantly suppressed the migration of NIH3T3 cells (p<0.01). Further, NIH3T3 cell migration was significantly reduced by NC siRNA-RKO CM to which a neutralizing anti-TGFβ1 antibody had been added (NC siRNA/Tβ1Ab; Fig. 3A). This did not occur when an IgG control antibody was added instead (NC siRNA/IgG; Fig. 3A). These findings suggest that the RKO cell-secreted factor(s) likely include active TGFβ1, since CRC cells are known to secrete this cytokine [20], [40] and the TGFβ1 neutralizing antibodies produced an effect similar to CM from RKO cells expressing the km23-1 siRNAs. Thus, our results suggest that suppression of NIH3T3 cell migration by km23-1-siRNA-RKO CM was likely due to a blockade of the paracrine effects of tumor cell-secreted TGFβ1.

As another measure of the paracrine, pro-oncogenic effects of TGFβ1, we investigated the effects of km23-1 knockdown in the RKO cells on NIH3T3 cell mitogenesis in co-culture studies. For these studies, RKO cells or their siRNA transfectants were co-cultured with NIH3T3 cells, prior to performing mitogenesis assays in the NIH3T3 cells, as assessed by [^3^H] thymidine incorporation [20]. As shown in Fig. 3B, for the EV and NC siRNA-RKO cells, NIH3T3 mitogenicity was enhanced compared to medium only (SM). In contrast, co-culture of km23-1 siRNA clone #1 or #5 RKO cells with the NIH3T3 cells significantly reduced the pro-mitogenic effects of the CRC cell-secreted factors on the fibroblasts (p<0.01). In addition, a neutralizing anti-TGFβ1 antibody was added to the NC siRNA-RKO co-cultures, similar to the CM studies in Fig. 3A. When these cells were co-cultured with the NIH3T3 cells (NC siRNA/Tβ1Ab), NIH3T3 cell mitogenesis was significantly reduced compared to co-cultures with RKO cells containing only IgG (NC siRNA/IgG; Fig. 3B). This finding suggested that the reduction in NIH3T3 mitogenesis we observed in co-cultures with RKO siRNA clone #1 and #5 cells was likely mediated by RKO cell-secreted cytokines such as TGFβ1. Thus, km23-1 can regulate fibroblast mitogenic responses through a paracrine effect of tumor cell-secreted factors such as TGFβ1, as revealed in co-culture studies.

### km23-1 knockdown inhibits cell migration and invasion of human CRC cells

**Figure 4:**
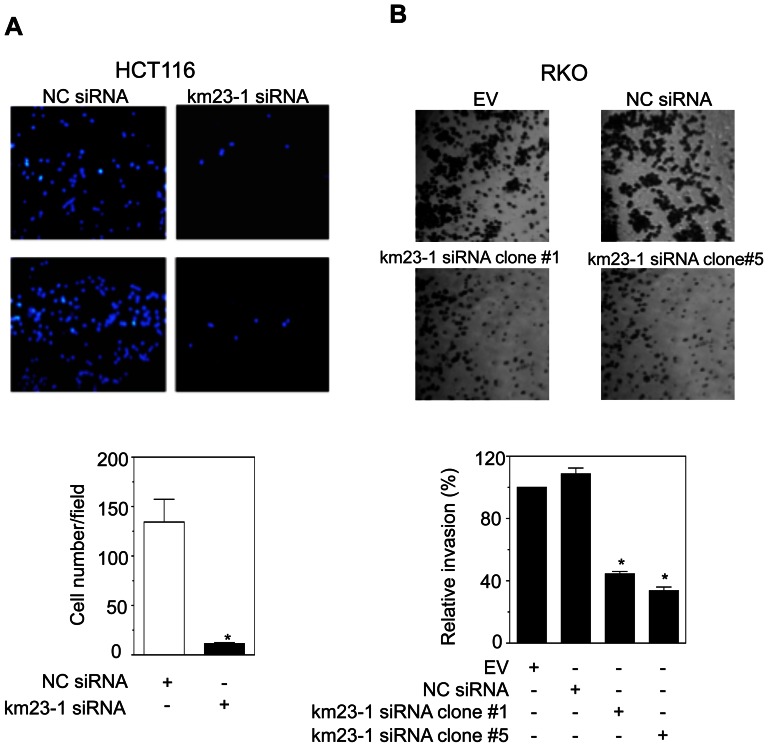
Depletion of km23-1 inhibits cell migration and invasion of human CRC cells. **A:** Transwell migration assays were performed as described in “Materials and methods.” Briefly, HCT116 cells stably transduced with lentiviral vectors expressing either piLenti-NC siRNA-GFP or piLenti-km23-1 siRNA-GFP were seeded into the upper wells of the Costar Transwell System (8-µm pore size polycarbonate membrane, 6.5-mm diameter), and the cells on the lower surface of the well after 24 h were fixed in methanol and stained with DAPI. **Top**, representative images of the lower surface of the membrane are shown (100× magnification). **Bottom**, the number of migrating cells of both NC siRNA- and km23-1-siRNA-HCT116 stable transfectants were counted under a fluorescence microscope and statistically analyzed. *p<0.01 compared to NC siRNA. **B:** Matrigel invasion assays were performed with the indicated RKO stable cells clones (clones #1, 5) for 24 h using EGF (20 ng/ml) as the stimulus as described in “Materials and methods.” Invaded cells were stained with 0.2% crystal violet. **Top**, representative images of the lower membrane surface from Matrigel are shown (100× magnification). **Bottom**, the number of invading cells for both NC siRNA and km23-1-siRNA HCT116 stable transfectants were counted under a light microscope and statistically analyzed. *p<0.01 compared to EV.

Since we have shown that depletion of km23-1 decreased various markers of a pro-invasive phenotype in human CRC cells (ie, ERK, TGFβ1), we examined the effects of km23-1 knockdown on cell migration and invasion of human CRC cells in Transwell and Matrigel assays, respectively. As demonstrated in Fig. 4A, depletion of km23-1 significantly inhibited cell migration of HCT116 cells compared to NC siRNA-HCT116 cells (p<0.01). Similarly, knockdown of km23-1 (km23-1 siRNA clone #1 and #5) significantly inhibited cell invasion of RKO human CRC cells compared to EV and NC siRNA-RKO cells (p<0.01) (Fig. 4B). Our results demonstrate that km23-1 is required for the cell migration and invasion of aggressive, KRAS- and BRAF-mutant human CRC cells, respectively.

### Depletion of km23-1 reduces Ezrin expression in human CRC cells

**Figure 5:**
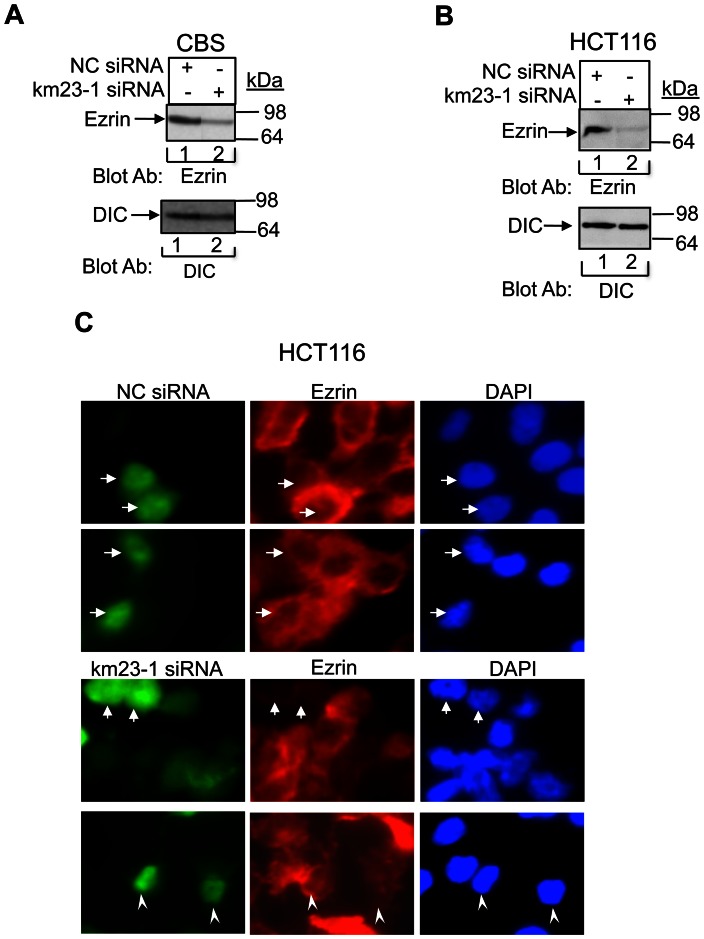
Depletion of km23-1 reduces Ezrin expression in human CRC cells. **A:** The cell lysates used for Fig. 1D were further subjected to Western blot analysis of Ezrin expression **(Top). Bottom**, DIC loading. **B:** HCT116 cells were infected with either pilenti-NC siRNA-GFP or the pilenti-km23-1 siRNA-GFP set. 24 h after infection, Western blotting was performed using the indicated antibodies. **Top**, confirms knockdown of endogenous km23-1. **Bottom**, DIC protein was assessed as a loading control. **C:** HCT116 stable pools that migrated through to the lower membrane in the Matrigel invasion assay were stained with an Ezrin Ab, followed by cy3-conjugated goat anti-rabbit IgG (red). DAPI staining permitted visualization of nuclei of individual cells (blue). GFP was used as a marker to designate cells transduced with siRNA (green). The cells were analyzed by an Olympus IX81 microscope at a magnification of 1000× with the appropriate filter sets.

Previous reports have shown that up-regulation of Ezrin is associated with epithelial tumor invasion and metastasis [17], [18], [51]. Since Ezrin represents another pro-invasion marker for human CRC [17], [51], we examined the effect of km23-1 depletion on expression of this cytoskeletal linker protein in two different CRC cell models, harboring distinct KRAS mutational events [ie, codon 13 (G13D) in HCT116 cells and a G12D mutation in CBS cells [24]]. As shown in Fig. 5, our results demonstrate that km23-1 knockdown reduced Ezrin expression in both CBS (Fig. 5A) and HCT116 (Fig. 5B) human CRC cells. Thus, km23-1 is required for another critical factor associated with the propensity of CRC cells to migrate and invade during tumor progression, despite the type of KRAS mutation that the cells contain. Also of significance with regard to CRC invasion is the ability of Ezrin to function as a plasma membrane-actin cytoskeletal linker at the leading edge of invading cancer cells [52]. Accordingly, we investigated the effect of km23-1 silencing on Ezrin immunofluorescence staining in HCT116 CRC cells that had invaded through a 3D matrix. As shown in Fig. 5C, km23-1 silencing reduced Ezrin staining compared to the NC siRNA cells (see arrows). While some cells depleted for km23-1 had scarcely detectable Ezrin staining, other cells did still express Ezrin, but at diminished levels (see arrowheads). Thus, while km23-1 silencing appears to reduce overall cellular expression of Ezrin (Figs. 5A, B), its depletion may also affect other functions at discrete areas of invading cells.

### Silencing of km23-1 does not affect cell viability in two KRAS-mutant CRC cell lines (HCT116, CBS), but it does reduce the growth of a BRAF-mutant CRC cell line (RKO)

**Figure 6:**
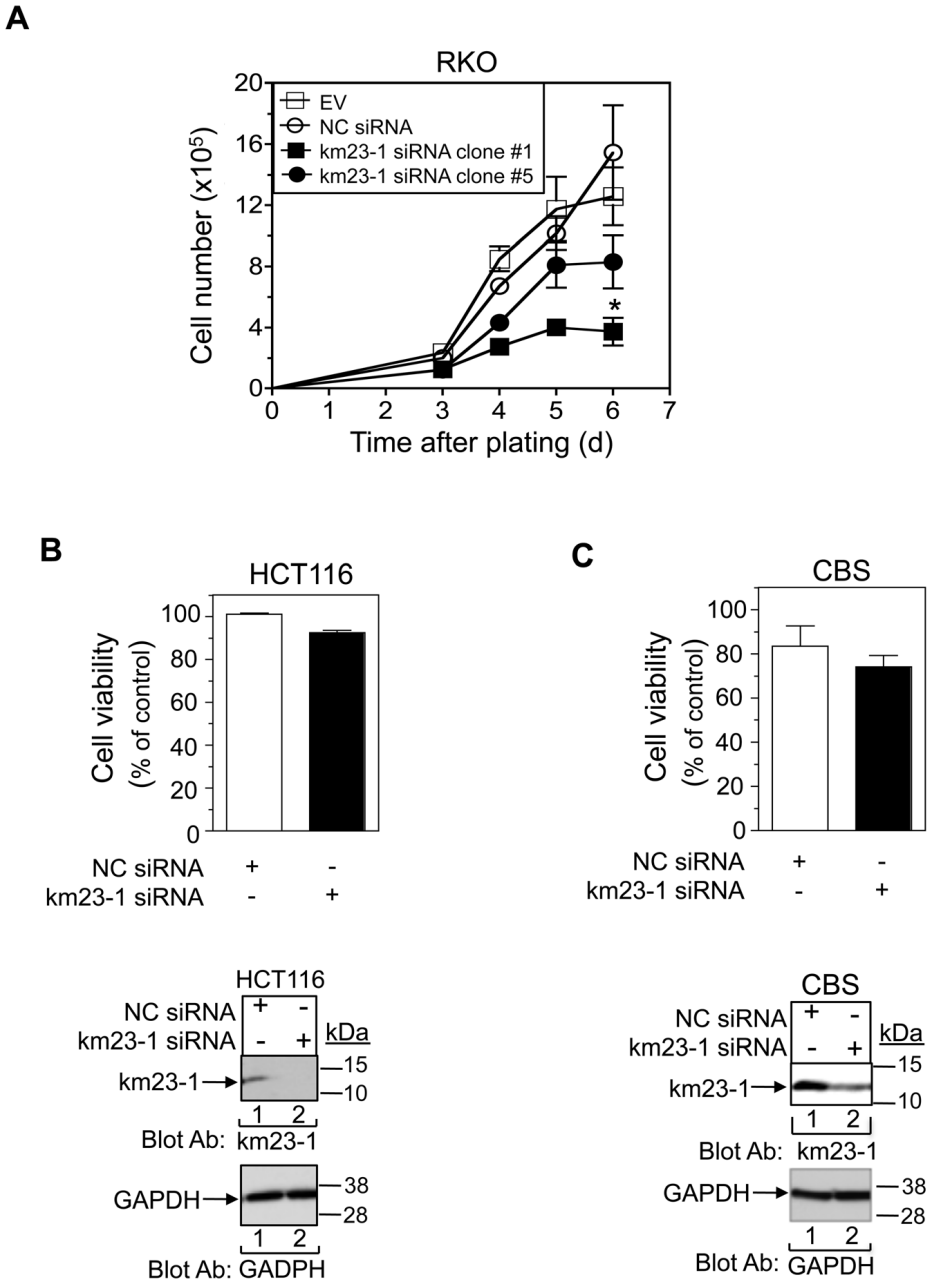
km23-1 silencing inhibits the growth of RKO cells, but not of HCT116 and CBS cells. **A:** RKO cell clones stably expressing EV, NC siRNA, or km23-1 siRNA [35] were plated and analyzed for cell number by trypan blue exclusion staining over the indicated days after plating. Mean ± SE (n = 3), *p<0.05 compared to the NC siRNA on day 6. **B: Top**, HCT116 human CRC cells stably transduced with either pilenti NC siRNA-GFP or pilenti km23-1 siRNA-GFP pools were subjected to XTT assays as described in “Materials and Methods.” Mean ± SE (n = 3). **Bottom**, HCT116 stable pools were grown and harvested for Western blotting to detect endogenous km23-1. GAPDH expression was used as a loading control. **C: Top**, similar XTT assays were performed in CBS cells stably transduced with either pilenti NC siRNA-GFP or pilenti km23-1 siRNA-GFP pools. **Bottom**, CBS stable pools were analyzed by Western blotting as for the bottom panel of **B**.

Since impaired cell viability may adversely effect cell invasion and migration, we investigated whether km23-1 knockdown affected the growth and viability of the CRC cells. As shown in Fig. 6A, we found that stable km23-1 depletion did reduce the growth of the BRAF-mutant RKO cells over the 6-day period analyzed. However, there were clonal differences in this regard, with clone #1 displaying a statistically significant decrease in cell growth (p<0.05), in comparison to clone #5, which only showed a slight inhibitory effect. In contrast to the RKO cells, km23-1 silencing had no effect on the viability of two KRAS-mutant human CRC lines (Figs. 6B, C). The growth inhibition upon depletion of km23-1 in RKO cells, but not in HCT116 or CBS cells, suggests that the signaling events which km23-1 controls may vary depending upon the oncogenic alterations that prevail in a given CRC model system, as well as on the availability of normal survival signals. Further, the absence of an effect of km23-1 depletion on cell viability in the HCT116 and CBS cells indicates that the anti-motility effects of km23-1 depletion are not secondary to effects on cell growth.

### Inhibition of km23-1 suppresses RKO tumor growth in vivo

**Figure 7:**
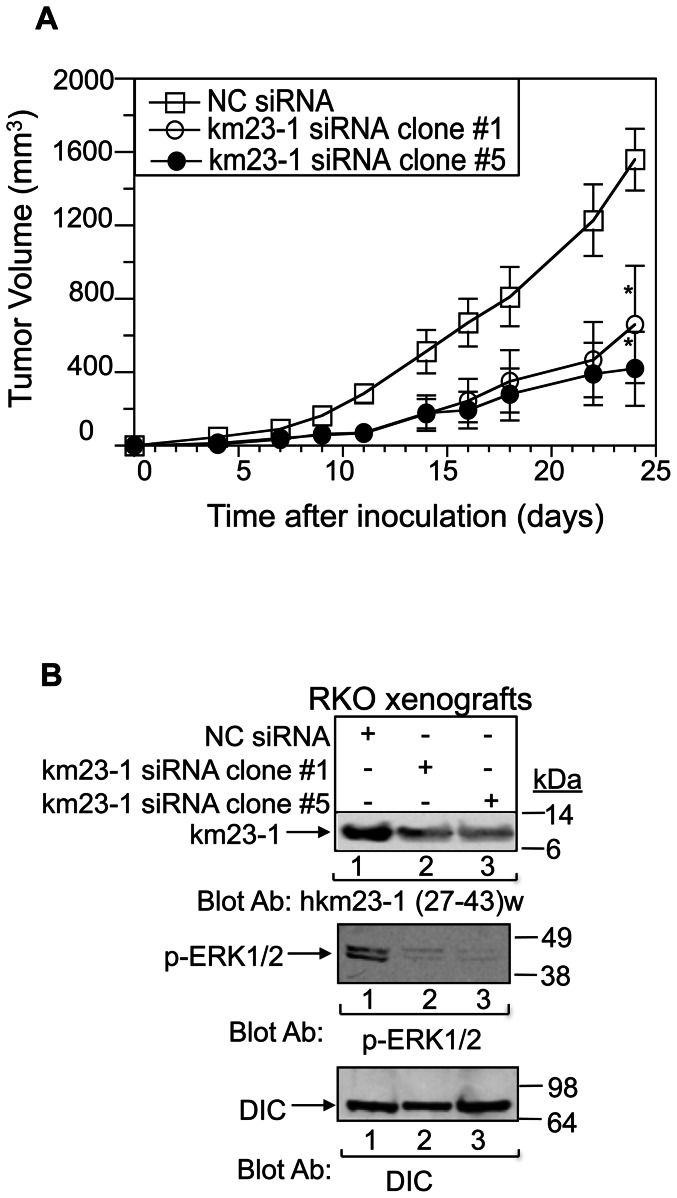
km23-1-siRNA blocks tumor growth of RKO cells *in vivo*. **A:** NC-siRNA RKO and km23-1siRNA RKO clone #1 and #5 cells (5×10^6^) were inoculated subcutaneously behind the right anterior forelimb of nude mice (n = 5). Tumor volumes were calculated as (length×width^2^)/2. *p<0.05 compared to NC, ANOVA. **B:** At the termination of the study, ERK activation and km23-1 knockdown in RKO xenografts were confirmed by Western blotting using the indicated antibodies. DIC, loading control.

Since we have shown that inhibition of km23-1 could block various ERK-mediated malignancy-associated events and is required for cell migration and invasion of human CRC cells, it was conceivable that km23-1 inhibition might also block tumor growth in vivo. To examine the effect of km23-1 depletion on tumor growth by human CRC cells in vivo, we performed in vivo tumorigenicity studies as described in the “Materials and methods.” Female athymic Balb/C nude mice were inoculated with NC siRNA-RKO cells or with km23-1siRNA RKO clones #1 and #5. As observed previously for RKO human CRC cells [53], mice formed tumors within 4d after inoculation (Fig. 7A). km23-1-siRNA RKO tumors grew at a significantly slower rate than the NC-siRNA RKO tumors. At 24d, the mean tumor sizes of km23-1-siRNA-RKO tumors were approximately 50% those of the NC-siRNA tumors. Thus, km23-1 knockdown caused a suppression of RKO tumorigenesis in vivo. In addition, knockdown of km23-1 reduced phospho-ERK expression in RKO xenografts (top panel, Fig. 7B), with km23-1 expression being inhibited throughout the experimental period. Thus, we show for the first time that knockdown of km23-1 inhibits the tumor growth of human CRC cells in vivo in association with reduced ERK activity.

## Discussion

ERK signaling plays multiple roles in the acquisition of a complex malignant phenotype. Specific blockade of the ERK pathway events results not only in anti-proliferative effects, but also in both anti-invasion and anti-metastatic effects in tumor cells [54]. In this report, we have shown that km23-1 depletion can block events known to be involved in cell invasion and tumor metastasis, such as activated ERK and high expression of Ezrin. We also describe for the first time that km23-1 is required for TGFβ1 secretion by human CRC cells, and that km23-1 knockdown can reduce the functional effects of CRC-secreted paracrine regulatory factors. More importantly, we demonstrate that km23-1 is required for tumor progression in vivo, as well as for the migration and invasion of human CRC cells. Thus, km23-1 may be a novel anti-cancer progression and motility target for colon cancer.

In accordance with the multifaceted nature of the km23-1 dimer [32], [33], [55], we have previously shown that siRNA knock down of km23-1 reduces TGFβ stimulation of TGFβ1 production in untransformed epithelial cells, partially through km23-1's Ras adaptor function [35]. In contrast, malignant cells have often lost TGFβ responsiveness and the associated tumor-suppressive functions, but still secrete TGFβ, which can assume paracrine, stimulatory functions with respect to tumor progression, invasion, and metastasis [26], [28]. TGFβ also plays an important role in tumor-stromal interactions during cancer progression and metastasis [23], [26], [28]. Thus, blocking the production of metastasis-promoting cytokines such as TGFβ is advantageous. Here we show that km23-1 depletion can decrease production of TGFβ1 in CRC cells that are deficient in TGFβ RII expression and contain BRAF or KRAS mutations. km23-1 inhibition also attenuated TGFβ1 promoter activity, AP-1 binding to the relevant TGFβ1 promoter site, as well as Elk-1 activity, effects that are required for TGFβ1 production [46] and play important roles in cell motility [15], [56]. In addition, km23-1 knockdown diminished the stimulatory effects of CRC cell-secreted factors (ie, TGFβ) on fibroblast migration and mitogenesis, whether the secreted factors were obtained from conditioned medium or released into fibroblast co-cultures. Overall, the TGFβ studies herein demonstrate that depletion of km23-1 is capable of blocking constitutive TGFβ1 production in human CRC cells, which, in turn, diminishes TGFβ's paracrine effects on cells in the tumor microenvironment.

Besides the paracrine effects of secreted TGFβ, TGFβ released into the tumor microenvironment can stimulate TGFβ-responsive cells to undergo EMT, which plays an important role in cancer invasion and metastasis [28], [57]. Here we have shown that knockdown of km23-1 inhibited the cell migration and invasion of human CRC cells, implicating km23-1 in the motility of invasive human CRC cells. The CRC cell models employed in our studies have KRAS (or BRAF) mutations [24], [45] and/or activated epidermal growth factor receptor (EGFR) pathways [58] , but they express comparatively low levels of EMT markers (ie, Snail1/2, Twist, and FOXC2 [59]), and their morphological features are indicative of an epithelial nature [59]. Along these lines, while colon cancer is generally considered to be a molecularly heterogeneous disease, two primary intrinsic subtypes that predict disease progression and recurrence have been identified [29]. These include a mesenchymal subtype that has undergone EMT, and an epithelial subtype that is associated with Ras activation, whether due to KRAS mutations or to activated EGFR pathways. While many tumor studies have focused on the EMT type [28], [60], [61], [62], here we have examined models of the other subtype. Since our current findings demonstrate that km23-1 depletion can decrease CRC migration and invasion in the non-EMT-correlated subtype of CRC, our data also indicate that inhibition of cell migration and invasion of human CRC cells can occur via mechanisms that do not involve EMT.

Besides the effects of km23-1 inhibition on the pro-invasion targets TGFβ and ERK, we also describe the effects of km23-1 knock down on the metastasis-associated target Ezrin. Of note, Ezrin expression levels are elevated in human CRC cancer cells, compared to normal tissues [17], [51]. Further, Ezrin is an ERK- and AP-1-induced target, which is known to be associated with malignancy and can be regulated by TGFβ [15], [63], [64]. Since we have shown that km23-1 silencing attenuates ERK and Elk-1 activity, as well as c-Fos/AP-1 binding, this pathway likely accounts for the reduced overall cellular expression levels of Ezrin reported herein. Thus, km23-1 may regulate Ezrin expression in a manner similar to the mechanism for regulation of TGFß1 expression, specifically with regard to the involvement of the AP-1 sites in their respective promoters [16], [47].

In addition to the increased overall expression of Ezrin in CRC, Ezrin provides a critical scaffold linkage between the plasma membrane and the actin cytoskeleton, thereby enhancing cell motility, invasion, and metastasis in many human cancers, including human CRC cells [18], [65], [66]. Depletion of this critical cytoskeletal linker was shown to decrease TGFβ and Ras/ERK signaling through novel mechanisms [4], [64], [65]. Further, mutations that affect Ezrin binding proteins have been shown to dominantly suppress both activation of TGFβ and FN production [63]. Here we have shown that km23-1 depletion in CRC cells reduces expression levels of this key scaffold protein, implicating km23-1 in the regulation of pro-migratory Ezrin complexes in the context of cell migration. Further, we demonstrate that km23-1 silencing can reduce Ezrin localization in CRC cells that have invaded through a 3D matrix, supporting a role for km23-1 silencing in the ability to prevent assembly of invasion-related Ezrin-scaffolded complexes.

In addition to Ezrin's functions in regulating growth factor signaling and in tethering F-actin to focal adhesions, it is an A-kinase anchoring protein (AKAP) that can target PKA to specific cellular compartments [67], [68]. Along these lines, we have shown that PKA can directly phosphorylate km23-1 on specific serine residues, which is required for TGFβ effects on downstream signaling of cAMP-responsive element (CRE)-dependent events (ie, FN) and Smad/activin-responsive element (ARE) activity [37]. Thus, km23-1 effects on Ezrin, as well as on other AKAP scaffolds, may involve PKA activity. This aspect of km23-1 regulation may be especially important for cell motility, as PKA activity has been shown to govern the continuous cycles of RhoA-mediated protrusion-retraction in migrating cells [69]. Further, we have already shown that km23-1 plays a role in RhoA activation and stress fiber formation [30]. Since km23-1 is a member of the GAMAD family in the human proteome (uniprot), it is structurally similar to p14, which binds to MEK partner 1 (MP1). The p14-MP1 complex can function as a MEK/ERK scaffold that regulates focal adhesion remodeling during cell spreading on FN [70]. Similar to p14, the km23-1 dimer likely provides an interface for binding of various scaffolded signaling complexes, permitting mutifunctionality at specialized regions of the cell. Further investigation will be required to address these additional complexities regarding km23-1 functions in cell migration and invasion.

Overall, our findings demonstrate for the first time that km23-1 regulates not only CRC cell migration, invasion, and tumor growth, but also critical markers of a pro-invasive phenotype, including TGFβ1, Ezrin, and ERK. We suggest that km23-1 may function in the assembly of key signaling complexes at critical subcellular regions associated with the invading edges of CRC cells. As such, km23-1 inhibitors may reduce the propensity for malignant CRC cells to invade, prior to frank metastasis. The ability of km23-1 to participate in such a wide range of biological activities associated with CRC cell progression and invasion suggests, further, that km23-1 inhibitors may be successful in the suppression of CRC metastasis. Analysis of the precise nature of the km23-1 complexes with AKAPs, actin-regulatory proteins [30], and the actin cytoskeleton at invasive sites will permit more accurate targeting of km23-1 to reduce the localized pools of activated signaling in these specialized regions of the cell.
